# The Relationship between Mucins and Ulcerative Colitis: A Systematic Review

**DOI:** 10.3390/jcm10091935

**Published:** 2021-04-30

**Authors:** Esther Bankole, Emily Read, Michael A. Curtis, Joana F. Neves, James A. Garnett

**Affiliations:** Centre for Host-Microbiome Interactions, Faculty of Dentistry, Oral & Craniofacial Science, King’s College London, London SE1 9RT, UK; esther.bankole18@imperial.ac.uk (E.B.); emily.read@kcl.ac.uk (E.R.); mike.curtis@kcl.ac.uk (M.A.C.); joana.pereira_das_neves@kcl.ac.uk (J.F.N.)

**Keywords:** mucin, MUC, ulcerative colitis, Inflammatory Bowel Disease, mucosa, intestine

## Abstract

Mucins are a family of glycosylated proteins which are the primary constituents of mucus and play a dynamic role in the regulation of the protective mucosal barriers throughout the human body. Ulcerative colitis (UC) is an Inflammatory Bowel Disease (IBD) characterised by continuous inflammation of the inner layer of the large intestine, and in this systematic review we analyse currently available data to determine whether alterations exist in mucin activity in the colonic mucosa of UC patients. Database searches were conducted to identify studies published between 1990 and 2020 that assess the role of mucins in cohorts of UC patients, where biopsy specimens were resected for analysis and control groups were included for comparison. 5497 articles were initially identified and of these 14 studies were systematically selected for analysis, a further 2 articles were identified through citation chaining. Therefore, 16 studies were critically reviewed. 13 of these studies assessed the role of MUC2 in UC and the majority of articles indicated that alterations in MUC2 structure or synthesis had an impact on the colonic mucosa, although conflicting results were presented regarding MUC2 expression. This review highlights the importance of further research to enhance our understanding of mucin regulation in UC and summarises data that may inform future studies.

## 1. Introduction

Mucin (MUC) genes encode a family of glycosylated proteins—mucins, which constitute the primary organic component of mucus [1]. The biophysical and biochemical properties of mucus are governed by mucin structure. Mucus is present on the surface of mucosal epithelial cells [1] and the role of mucus and the mucosal epithelia in health and disease is dynamic. Mucosal surfaces act as the first line of defense against pathogenic microbes and foreign particles [2]. In addition to forming a physical barrier which prevents the entry of pathogens into mucosal epithelia, mucus, synthesised by goblet cells, is a lubricant which protects the epithelia from damage and retains cellular hydration [2,3]. Mucins are essential components of mucus and the mucosal defense system, regulating the absorption of nutrients and offering protection against infections that may lead to mucosal disease [1,4,5]. Currently the expression of 21 MUC genes (mRNA and/or protein) have been identified at various mucosal sites throughout the body [6].

Mucins are characterised by their central domain, which is composed of a protein backbone containing Proline, Threonine and Serine (PTS) rich Variable Number Tandem Repeat (VNTR) sequences, also termed tandemly repeated polypeptides. The PTS rich tandemly repeated polypeptides have extensive *O*-linked glycans [4,7,8]. *O*-linked glycans are attached within the PTS rich regions as glycosidic bonds between the side chain hydroxyl groups of serine and threonine residues and the sugar *N*-acetylgalactosamine (GalNAc) [2,3]. *N*-linked glycans on the other hand tend to be found towards the terminal domains of mucins [4,9].

The length and number of PTS rich VNTRs can be used to distinguish mucins from each other [4,5,10]. For example, structural analysis of MUC1 has revealed an uninterrupted PTS region whilst MUC2 has two PTS-regions [10]. Differences in the structures of mucins, particularly in the assembly of their domains are reflective of their functions, which are dependent on the location of the mucosal epithelia from which they originate within the body [2]. For this reason, mucins can be described as tissue specific molecules [2,5,10]. There are two major groups of mucins: secretory mucins and membrane-associated mucins [9,11,12]. Secretory mucins can be divided into the subgroups secreted soluble mucins (or non-gel forming) and secreted gel forming mucins. Mucins within the same group share distinctive conserved domains.

Membrane-associated mucins such as MUC1 have a monomeric structure and contain distinct transmembrane domains, which enable them to be incorporated into the apical cell surface of epithelia, as well as a short cytoplasmic tail and a Sea urchin-Enterokinase-Agrin (SEA) domain [2,4,13]. The SEA domain joins the tandemly repeated polypeptide region to the transmembrane domain. Some of the membrane-associated mucins also have an Epidermal-Growth-Factor (EGF) like domain [10,14]. Instead of the SEA domain, MUC4 has a Nidogen-AMOP domain [10,15]. Secreted gel forming mucins such as MUC2 have an oligomeric structure which appears as a layered network of monomers bonded together through disulphide bridges whereas secreted soluble mucins such as MUC7 are much smaller and appear monomeric [2,10,16]. The gel forming secreted mucins can be distinguished by their cysteine rich domains and their ability to form von Willebrand factor like C and D domains [4,10,17]. However, the secreted soluble mucins (MUC7, MUC8, MUC9) are not as well characterised as secreted gel forming mucins.

In order for the mucosal barrier to maintain protection against external pathogens, mucins are required to take part in various pathways [2]. Mucin degradation and turnover are essential processes that help to balance the equilibrium between the biosynthesis of mucosal membranes and the secretion and breakdown of mucus gel, which in turn regulates the protective mucosal barrier [2]. To understand the significance of mucin degradation and to explore how degradation occurs, the structure of mucins and their ability to form interactions with microbes must be understood [2,3]. It has been shown that mucins have a dualistic role within the protective barrier; mucins are able to prevent the entry of pathogenic organisms/substances by adhering to them whilst also forming interactions with commensal bacteria via glycosylated attachment sites [3,18,19]. It must be noted that by forming these interactions with bacteria, mucins provide an energy source for bacteria capable of mucin degradation [2,3]. Binding of mucins to bacteria thereby facilitates their breakdown by mucin degrading enzymes that cleave glycosidic bonds; thus, it can be said that mucins also act as substrates for mucin degrading bacteria [2,3]. Although mucin degradation is essential, in some cases, the equilibrium between mucin degradation and mucosal biosynthesis becomes perturbed leading to a shift in the composition of the microbiota [2,3]. In these circumstances, mucin degradation may be associated with the primary stages of the development of mucosal diseases, such as Inflammatory Bowel Disease (IBD).

IBD is a term used to describe diseases characterised by chronic intestinal inflammation such as Crohn’s Disease (CD) and Ulcerative Colitis (UC) [20,21]. Several differences exist between CD and UC, including the distribution and spread of inflammation. In UC, inflammation is continuous, uniform and is restricted to the superficial layers of the large intestine [20]. In contrast, in CD inflammation is observed in any region of the gastrointestinal tract (GI tract) from the mouth to the anus, is discontinuous and occurs in all of the layers lining the intestines [20,22]. The global burden and incidence of IBD continues to rise, with more than 6.8 million cases of IBD worldwide reported in 2017 [23]. The underlying aetiology of IBD remains unclear. IBD is multifactorial; the incidence of IBD can be associated with immune disorders, genetics, environment and interactions with microbes [20]. Recent research has suggested that perturbation and dysfunction of the intestinal mucosal membrane is related to the pathogenesis of IBD [3].

Analysis of biopsy samples of the colonic mucus lining shows the presence of two sub-layers: an outer layer that acts as a lubricant and an inner or adherent layer that acts as a selective barrier. This adherent layer limits direct contact between the luminal contents (including the microbiota) and epithelial cells and is the site of expression for the majority of mucins detected in the colon [3,24]. MUC2 is the most prominently expressed mucin in the colon of both healthy individuals and individuals with UC [3,25]. However, biopsy samples have revealed that the adherent mucus layer of patients with UC is thinner, particularly in areas of inflammation [26] As described above, goblet cells are responsible for the synthesis and excretion of mucins such as MUC2 [25] and it has been suggested that the reduction of goblet cells associated with UC mucosal perturbation is related to a reduction in the expression of mucins. This is associated with a loss of integrity of the mucosal barrier, which leads to infiltration of microbes into the mucosa, increasing the contact between the epithelia and microbiota, which promotes further inflammation [24]. However, the significance of aberrant mucin expression in UC remains uncertain.

This review aims to compile recent data regarding the relationship between mucin activity and UC, in order to provide a clear, concise summary which can be used to inform further studies in this area of research. In addition, the collated information is used to identify any possible correlations between mucin expression levels and mucin degradation within the colon of UC patients. An improved understanding of the role that mucins and their turnover play in the protective barrier at mucosal membranes will be important in clarifying their potential involvement in the prevention of mucosal disease such as UC and CD.

## 2. Materials and Methods

### 2.1. Review Questions

1. Does mucin expression and activity change in patients with UC compared to healthy controls?
2. How do changes in mucin expression effect mucus structure?
3. What impact do these changes have on host-microbiome interactions and inflammation?

### 2.2. Search Strategy

An electronic search was performed in the following databases up until December 2020: PubMed, Ovid MEDLINE, Cochrane CENTRAL (TRIALS) and Web of Science. The search terms used to address the objectives were:

- Colitis AND mucin *
- (IBD OR Inflammatory Bowel Disease) AND mucin *
- (IBD OR Inflammatory Bowel Disease) AND mucin * degradation
  To optimise the retrieval of articles in databases, Boolean operators were used (AND, OR). Using ‘AND’ allows for citations containing all search terms to be retrieved, whereas using ‘OR’ can produce citations containing at least one of a group of search terms. Therefore, ‘OR’ was used here where words were similar/related e.g. IBD and Inflammatory Bowel Disease. Mucin was also truncated (truncation is indicated by Asterix – *) in order to find citations including mucin and all words with the stem ‘mucin’ e.g. mucin, mucins, mucinase.

### 2.3. Study Selection and Items Collected

To aid with the screening process, articles found during the search process were imported from databases to Covidence (Veritas Health Innovation, Australia), the Cochrane approved systematic review manager. Duplicates were identified and removed automatically using Covidence and the remaining articles were moved to a panel for screening. Titles and abstracts were screened to determine whether articles matched the study criteria. Articles that matched study criteria were moved to another panel for full text screening and in accordance with the inclusion criteria, eligible studies were chosen to be included for subsequent analysis and data extraction. The inclusion and exclusion criteria were entered into the Covidence programme, so as papers were excluded the reasons for exclusion were recorded for each paper. Following inclusion of relevant articles, citation chaining was carried out. Forwards citation chaining (to identify articles that cited an included paper and that were also relevant to the systematic review search questions) was carried out for each included article by searching article titles in Google Scholar and using the ‘cited by’ feature of the Google Scholar search. Backwards citation (to identify articles that an included paper cited and that were also relevant to the systematic review search questions) was carried out for each included article by manually scanning the references listed in included articles. Additional articles identified through citation chaining were imported directly into the data extraction panel in Covidence. Data were extracted from studies using the data extraction tool within Covidence, extracted data was summarised based on identification (Author, Country), methods used (study design, experiments/investigations), population (study size, study groups included), outcomes (key findings, conclusion from study). 

### 2.4. Inclusion & Exclusion Criteria

The inclusion criteria were articles where mucin mRNA and/or mucin protein expression had been studied and recorded in a cohort of patients with colitis/healthy controls; articles which assessed mucin structural changes in patients with colitis; articles which investigated mucin expression in reference to inflammation, disease severity and maintenance of the colonic mucosal epithelia; and articles published between 1990 and 2020. Review papers and meta-analysis reports, conference abstracts, doctoral theses, opinion articles, book chapters and letters to editors were excluded from further analysis. Likewise, articles not written in English; where colitis was primarily investigated using cell lines or animal models (experimental colitis-no human samples); only focused on colitis (mucin expression not investigated); where no controls were included in the study; if there was insufficient data; and if the full article could not be accessed. Clinical case studies were not specifically excluded, Cochrane CENTRAL a database of published clinical trial articles was used as part of the search strategy; however, papers were excluded based on the exclusion criteria stated (none of the clinical case studies identified through the search explored the relationship between mucins and colitis). Subsequently no clinical case studies could be included.

### 2.5. Study Quality Assessment

All included articles were peer-reviewed. Risk of bias was assessed as part of the appraisal stage during data extraction. A risk of bias scoring system was designed for this systematic review in order to rule out common forms of bias which are encountered in scientific research. The criteria assessed the impact of sample size, sample variability, selection bias, comparability of sample groups (patient samples and controls), reproducibility of investigations and reporting bias. Study quality was ranked on a scale from 0 (low quality) to 6 (high quality).

### 2.6. Data Synthesis

The focus of this review was the relationship between mucin activity and colitis, and therefore a narrative account of findings from the included studies detailing the mucins analysed and the fate of mucins during colitis was recorded. Findings and data from studies were tabulated. Quantitative synthesis of results using meta-analysis was not performed because the presentation of quantitative analysis (methods used for quantitative analysis and units) varied substantially across articles. Therefore, procedural bias was eliminated from this review by omitting meta-analysis.

## 3. Results

### 3.1. Search Results

**Figure 1:**
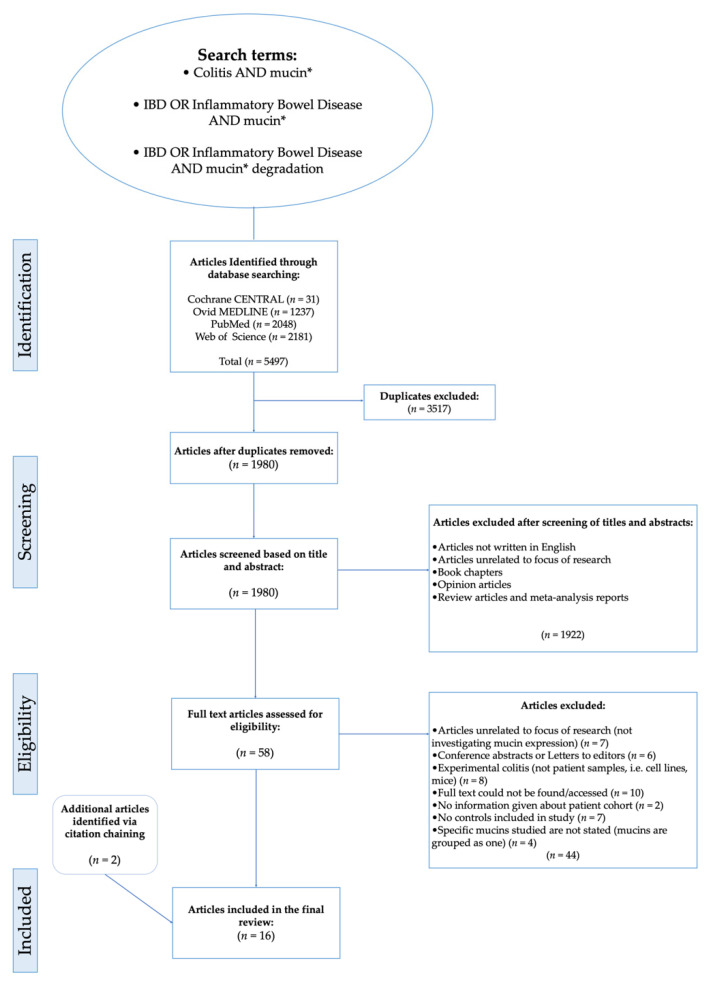
Flow chart showing steps of search process leading to the inclusion of articles for analysis. Boolean operators and search modifiers were using to optimise the retrieval of articles in the search strategy: ‘AND’ allows for citations containing all search terms to be retrieved, OR’ can produce citations containing at least one of a group of search terms. Therefore, ‘OR’ was used here where words were similar/related e.g., IBD and Inflammatory Bowel Disease. Truncation as denoted by asterix (*) was used to find citations including words with the same stem e.g. mucin, mucins, mucinase.

Figure 1 details a flowchart of the study selection [27]. A total of 5497 articles were identified by conducting searches on databases (Cochrane CENTRAL = 31, Ovid MEDLINE = 1237, PubMed = 2048, Web of Science = 2181). A total of 3517 duplicates were removed, then titles and abstracts of the remaining articles were screened. Following the screening process, 1980 articles were rejected based on the exclusion criteria because they were reviews, book chapters, opinion articles, not written in English or because the focus of the articles was unrelated to the aim and topic to be addressed in this systematic review. For the next stage of the selection process, a full text eligibility assessment was carried out on the 60 articles that remained after the initial screening process. Employing the eligibility criteria, 44 articles were excluded from further analysis and 14 that met the inclusion criteria were identified for subsequent data analysis.

**Figure 2:**
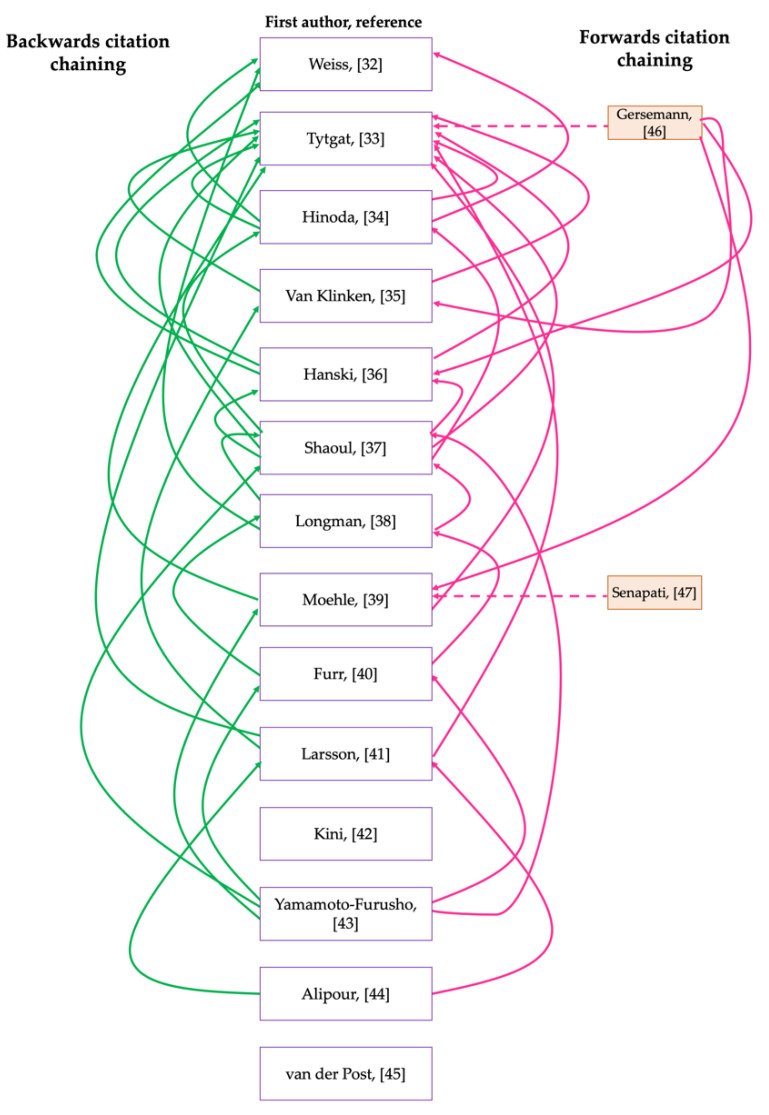
Citation network showing the output data of citation chaining process using key articles that were included for analysis following database searches and screening. Backwards citation chaining output data is presented on the left-hand side, here an arrow is pointed from the key articles used for citation chaining towards an article that the key article cites. Forwards citation chaining output data is presented on the righthand side, here an arrow is pointed towards the key article used for citation chaining from an article that has cited the key article. Dashed arrows are used to highlight articles that were identified by citation chaining.

Ten articles were excluded because the full text was inaccessible, therefore, assessment for eligibility criteria and subsequent analysis would not have been possible. Seven articles were also excluded because the studies did not include controls. Others were excluded because instead of including control groups, they compared mucin expression in the colonic mucosa of patients with UC to that of patients with CD. For example, Dorofeyev et al. [28] observed that in UC, mucin gene expression decreased in severe disease and the reverse of this was noted for CD. Similarly, Hensel et al. [29], which explored the differential expression of MUC1 and MUC2 in pediatric patients with IBD, was excluded; although a control cohort was included in this study, the findings were presented as differences between the expression of mucins at different severities (inflamed/non-inflamed) of CD and UC, while these studies are informative for our understanding of characteristic differences within the intestinal microenvironment of patients with UC and CD, the differences that may exist between healthy controls and patients with IBD are inconclusive from the findings presented. Therefore, the relationship between UC and mucins could not be accurately analysed and elucidated. Although some studies produced noteworthy results relating to the fate of intestinal mucin genes during colitis, they were excluded from further analysis because mucin gene expression was not investigated or documented. For example, Swallow et al. [30] determined that MUC2 allele length is not associated with disease pathogenesis, whilst Kyo et al. [31] detected rare MUC3 alleles in UC patients which they suggested may be associated with disease pathogenesis. In addition to the articles included through database searching [32,33,34,35,36,37,38,39,40,41,42,43,44,45] and screening, 2 more papers were identified through citation chaining [46,47] and included in the analysis group (Figure 1 and Figure 2). The citation network generated using output data from the citation chaining process highlights the connections between the included studies. The more recent studies cited some of the older studies (Figure 2).

### 3.2. Overview of Studies Selected for Analysis

The final 16 included articles were all peer-reviewed case–control studies, published between 1990 and 2020, assessing the role of specific mucins during UC by carrying out various investigations using resected biopsy samples from a cohort of patients (Table 1). 50% (*n* = 16) of the studies were conducted in European countries, whilst 31% (*n* = 16) were conducted in North America and 19% (*n* = 16) in Asia. None of the identified studies were performed in African or Southern American countries. 44% of the studies were published in the last 10 years.

Three of the included studies [37,40,44] were carried out using specimens resected from individuals with paediatric IBD. Eight articles focused solely on the relationship between MUC2 and colitis, whilst five articles studied MUC2 and a selection of other mucins in colitis. Three of the included articles did not study MUC2 at all, one investigated MUC1 [40], whilst the another investigated MUC12, MUC16 and MUC20 in UC [43] and finally one investigated MUC17 [47]. In order to create a timeline of the findings and to understand how the findings from different studies may be related to each other, a table listing the included studies in chronological order was produced (Table 2).

### 3.3. Critical Appraisal of Included Studies

**Figure 3:**
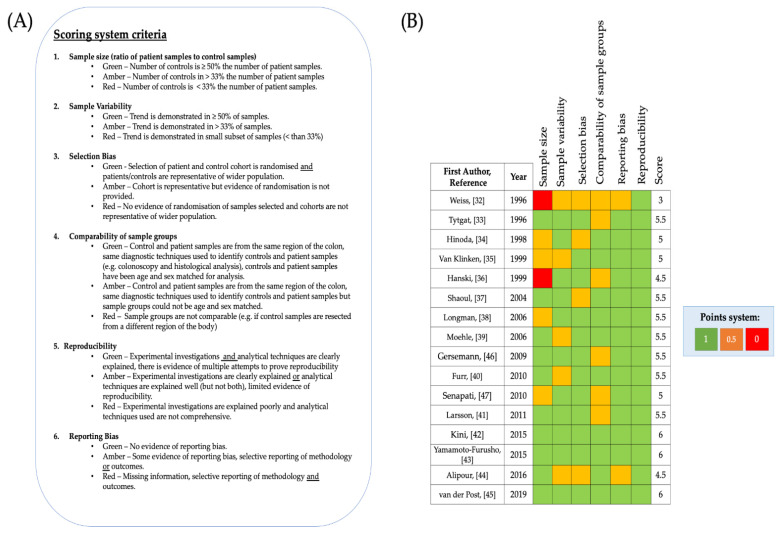
Quality assessment of included studies (**A**) Scoring system used with categories and information outlining how scores were allocated. (**B**) List of included studies and scores allocated to each included study using the quality assessment scoring system is shown.

The 16 included studies were assessed for bias before further analysis was carried out. Widely used systematic review scoring systems, for example the Cochrane risk of bias scoring system [48] have been designed to assess risk of bias in randomised clinical trials but were incompatible for this systematic review. Therefore, a specific scoring system was designed for this review. The aim of this scoring system was to rule out and take account of, if present, common forms of bias which are encountered in scientific research, in particular in case-control studies. Studies were scored Green-1, Amber-0.5, Red-0 for each category of the scoring system. The criteria for the system consisted of seven categories, and consequently a maximum of seven points could be awarded (Figure 3A). Studies with a larger number of specimens improve the ability to recognise and understand trends Therefore, to determine whether trends were random or consistent both sample size and sample variability were scored for all included articles. In case-control studies big differences between samples from individuals in the case group (which is individuals with UC in the included studies) and controls can lead to over-representation or under representation of results, therefore sample size was assessed as the ratio of patient samples to control samples. Another important factor to consider for case- control studies is the comparability between controls and patient samples, comparability was assessed in terms of the sample type and with reference to patient characteristics (Figure 3A). When considering the reliability of data, it is also important to determine the analytical and biological reproducibility of investigations, and so this was also assessed. Although the Cochrane risk of bias scoring system was not used, selection bias and reporting bias can still occur in scientific investigations and therefore these categories were retained for the new scoring system. According to this quality scoring system, the final selection of studies was considered to be high quality (Figure 3B), with the lowest score being 3/6 and three articles scoring 6/6. The mean number of participants with UC (patient cohort) across all included studies was 28, whilst the mean number of control participants across all studies was 16. Weiss et al. [32] and Hanski et al. [36] scored 0 for sample size because the ratio of their controls to patient samples was 1:8 (12.5%) and 13:70 (18.6%) respectively. The pitfall of the majority of the studies was that the size of their control cohort was less than 50% the size of the control cohort [32,34,35,36,38,47] and/or the comparability of patient samples to control samples was limited [32,33,36,41,46,47] although bias arising from sample variability [32,35,39,40,44] and/or selection bias [32,34,37,44] was recorded for a few of the studies. Studies scored 0.5 for comparability between samples if insufficient details were provided about the location of the intestinal/colonic mucosal tissue resected in healthy controls in comparison to the patient cohort and if there was insufficient information provided pertaining to the age ranges of samples from individuals in the control and/or the patient cohort. Weiss et al. [32] scored 0.5 for comparability between sample groups because the control sample tissue they analysed was resected from the small intestine, although UC can affect the ileum, the article does not state whether the UC samples they analysed were also resected from the ileum. Since the structure of mucosal layer in the small intestine and colon have slightly different characteristics [3] and UC rarely affects the small intestine Weiss et al. [32] also scored 0.5 for selection bias. Similarly, Alipour et al. [44] scored 0.5 for selection bias, since they analysed UC samples and control samples from the terminal ileum and the other manuscripts analysed samples from the colon, which is the main organ affected by UC. On the other hand, Van Klinken et al. [35] scored full points for comparability because all samples were resected from individuals in a similar age range and were all sex matched. However, since samples were all obtained from men (no women in patient or control cohort) in the study conducted by Van Klinken et al. [35], limited sample variability exists in this study. Weiss et al. [32] scored 0.5 for sample variability and reporting bias because the results presented were from one biopsy specimen and it was not specified in the paper whether the findings were consistently observed in a significant number of the collected specimens. Alipour et al. [44] scored 0.5 for sample variability and reporting bias, for the same reasons. Likewise, Furr et al. [40] scored 0.5 for sample variability because only 46% of UC specimens were successfully stained for analysis, but since Furr et al. [40] reported this sample variability, the study was deemed to have no evidence of reporting bias. Since biopsy specimens were resected from UC patients during routine colonoscopy and patient identification was blinded from researchers carrying out investigations, most studies were considered to have no selection bias. Control specimens across the studies were generally resected from individuals with no known history of UC undergoing diagnostic colonoscopy (shown to be negative for IBD) or screening for polyps. It must be noted that although the control samples included across the studies were resected from individuals with no history of IBD, some of the controls were taken from patients with other pathologies (Table 1). Several of the included studies used colonic mucosal tissue resected from patients with benign polyps, irritable bowel syndrome or diverticulosis/diverticulitis [32,35,39,43,44]. On the other hand, Hinoda et al. [36] used colonic mucosal tissue resected from patients post-mortem as control samples. For this reason, Hinoda et al. [34] was scored 0.5 for both selection bias and comparability of sample groups. Shaoul et al. [37] also showed some evidence of selection bias, as they reported that their control samples were taken from a histology library, which they did not justify (there is no information given about the patients that the tissue was resected from) However, Shaoul et al. [37] state that the control samples were from age-matched individuals, this increases the comparability between the sample groups.

### 3.4. MUC2 Expression in Colitis

MUC2 expression was examined at the nucleic acid and protein level. Eight studies investigated *MUC2* mRNA levels in UC in comparison to controls, nine studies investigated MUC2 protein expression levels. Van Klinken et al. [35] and Tytgat et al. [33] both recorded total MUC2. Van Klinken et al. [35] defined total MUC2 as the sum of the detected radiolabelled and non-radiolabelled MUC2 protein. In contrast, Tytgat et al. [33] defined total MUC2 as the sum of MUC2 precursor and mature, fully glycosylated intracellular MUC2 protein and adherent MUC2 protein. It must also be noted that, the methodological techniques employed to measure MUC2 expression varied across the studies depending on the purpose of the study, as highlighted in Table 2. The majority of the studies used in situ hybridisation and/or immunohistochemistry to detect MUC2 mRNA and/or MUC2 protein respectively. However, two studies also employed RT-PCR [39,46] to quantify gene expression by measuring *MUC2* mRNA levels.

#### 3.4.1. MUC2 mRNA Expression

Several of the studies found that *MUC2* mRNA expression levels were unaffected by UC (Figure 3A). Weiss et al. [32] were the first study to use in situ hybridisation to examine whether there were any changes in the cellular distribution of MUC2 as a result of IBD. Concordant with published data, through histological analysis, Weiss et al. [32] observed that *MUC2* mRNA was expressed in goblet cells of normal colonic tissue. The pattern of distribution of *MUC2* mRNA remained the same in colonic tissue from individuals with UC and the same study reported no apparent differences in the expression of *MUC2* mRNA between healthy controls and UC. Furthermore, Tytgat et al. [33] quantified *MUC2* mRNA levels and found that there were no statistically significant differences between the average levels of *MUC2* mRNA in UC samples in comparison to controls. To ensure validity of their results, prior to quantification Tytgat et al. [33] carried out control experiments to assess the size and condition of biopsy specimens and to test the viability of the anti-MUC2 antisense probes. Hanski et al. [36] also found no changes in *MUC2* mRNA levels between UC samples and controls. Contrary to Weiss et al. [32], Longman et al. [41] observed differences in the cellular distribution of *MUC2* mRNA between UC and controls and reported that an increase in disease severity was associated with a reduction in *MUC2* mRNA expression in goblet cells. Similarly, Alipour et al. [44] showed that there was a significant reduction in the mean number of goblet cells expressing *MUC2* mRNA and stated that this was related to aberrations in the intestinal epithelia in UC. Interestingly, Gersemann et al. [46] reported that a reduction in goblet cell differentiation led to the attenuation in the induction of *MUC2* mRNA synthesis in inflamed samples (Active UC) in comparison to non-inflamed samples, whilst they also observed an increase in *MUC2* mRNA when comparing both noninflamed and inflamed UC samples to controls. Whilst, Moehle et al. [39] reported that *MUC2* mRNA levels were downregulated in UC, they suggested that this observation may be a result of interpatient variations of mRNA expression levels between samples within study groups (UC and controls).

#### 3.4.2. MUC2 Protein Expression

After analysing the findings associated with *MUC2* mRNA expression levels, the findings associated with MUC2 protein expression levels were investigated (Figure 3B). In the study conducted by Tytgat et al. [33], when MUC2 precursor (identified as a mass of 600 kDa) biosynthesis and total MUC2 levels were quantified; the mean values were reduced significantly in samples obtained from patients with active UC in comparison to patients with UC in remission and controls. The study revealed that although these parameters were also decreased in patients with UC in remission, the values were not decreased to the same extent as in patients with active UC and the values were much closer to the values observed for controls, it was therefore suggested that changes in the level of MUC2 biosynthesis may be associated with inflammation and disease severity. Tytgat et al. [33] aimed to understand the regulation of MUC2 expression in UC; based on their findings, they proposed that MUC2 expression is regulated at the translational level. They observed that there was a direct correlation between the reduction in total MUC2 levels and the reduced synthesis of MUC2 precursor, which led them to propose that in active UC translation of *MUC2* mRNA is inefficient. Tytgat et al. [33] noted that further investigations are required to distinguish whether this finding is exclusive to UC or whether it occurs as a result of colonic inflammation independent of UC. Hinoda et al. [34] also demonstrated that MUC2 protein expression decreases in active UC, highlighting that it remains unknown whether the alteration leads to the pathogenesis of UC or whether the alteration occurs during the pathogenesis of UC. Interestingly, van der Post et al. [45] determined that structural components of the colonic mucosa, including MUC2 were reduced in regions without inflammation as well as inflamed regions of UC samples. This suggests that the reduction of MUC2 in UC is independent of and may precede inflammation. In agreement with this, when Kini et al. [42] investigated the relationship between the expression of colonic stem cells within non-inflamed regions of the lower crypt, they recorded that a 2-fold decrease in stem cells correlated with a reduction in MUC2 protein expression. Kini et al. [42] suggested that this reduction of MUC2 protein precedes inflammation and may be related to a reduction in the differentiation of goblet cells. However, Kini et al. [42] also proposed that the reduction in MUC2 protein is dependent on signalling pathways at different colonic niches, since the study also found no change in goblet cell markers at the upper colonic crypt in UC patient samples in comparison to control samples. Hanski et al. [36] suggested that changes in MUC2 protein expression may occur as a result of a long-term aberration in the post-transcriptional modification of MUC2. Although Hanski et al. [36] observed no changes in *MUC2* mRNA levels between UC and control samples, the study found an increase in the level of MUC2 protein in active UC compared to UC in remission and controls. Thus, Hanski et al. [36] proposed that the increase in MUC2 protein may be related to an inflammatory pathway which leads to rapid aberrant post-transcriptional modifications prior to translation of *MUC2* mRNA to MUC2 protein. The resulting proteins observed by Hanski et al. [36] were hypoglycosylated and/or poorly sulphated. Similarly, Van Klinken et al. [35] reported that MUC2 protein levels were reduced in active UC because MUC2 was under sulphated during synthesis and the reduced sulphate incorporation renders mucins more susceptible to degradation. Although Shaoul et al. [37] did not record changes in MUC2 protein expression levels, they reported that inflammatory damage resulted in the expression of hypoglycosylated MUC2 protein in what appeared to be immature goblet cells. Larsson et al. [41] also recorded no significant differences in relative mean amounts of MUC2 protein between UC patients and controls.

#### 3.4.3. Goblet Cells and MUC2

The included studies also explored whether differences existed in goblet cells in the colonic mucosa of UC patients compared to controls. Tytgat et al. [33] proposed that during active UC goblet cells could not efficiently synthesise MUC2 protein in comparison to during remission or in absence of UC. A change in efficiency of MUC2 protein production in active UC was also described by van der Post et al. [45]. In their study they performed microbial challenge of goblet cells by stimulating samples with the bacterial synthetic lipopeptide TLR2 ligand, Pam3CysSerLys4 (P_3_CSK_4_). They found that in active UC this led to the attenuation of goblet cell secretory action, and therefore a reduction in secretion of MUC2, and suggested this contributes to the structural weakening of the colonic mucosa. Hinoda et al. [34] on the other hand, showed that the presence of aberrant (poorly differentiated) goblet cells in active UC results in decreased MUC2 protein production. Concordant to this, Gersemann et al. [46] observed a reduction in the percentage of goblet cells in patients with UC when compared to controls. By examining the mRNA expression levels of goblet cell differentiation factors; Helix-loop-helix protein (hATH-1) and Krüppel-like factor 4 (KLF4), Gersemann et al. [46], proposed an association between impairments in goblet cell differentiation and reduced mucin synthesis in patients with UC. The study found that goblet cell differentiation and subsequent mucin synthesis is particularly reduced in inflamed UC samples. Kini et al. [42] also investigated the role of goblet cell differentiation marker KLF4, however, they noted that the relative expression of KLF4 in UC patient samples was not significantly different from controls. Kini et al. [42] identified differences between the differentiation of goblet cells in the lower crypt and upper crypt of colonic biopsy tissue. Although Kini et al. [42] noted no significant changes in the upper crypt, they reported a reduction in stem cells and enteroendocrine cells in the lower crypt, which may be involved in goblet cell differentiation and synthesis of MUC2. Whilst, Shaoul et al. [37] demonstrated that in samples from paediatric UC patients, MUC2 protein levels were preserved (unaltered). However, the protein was expressed in a hypoglycosylated form in secretory granules of cells, which were not phenotypically goblet cells, but which may be immature goblet cells [37]. An alteration of MUC2 protein glycosylation was also reported by Larsson et al. [41], who stated that there were two glycan profiles of MUC2. In the study Larsson et al. [41] noted that MUC2 protein of patients with active UC were composed of smaller glycans than in UC in remission and controls. The study [41] also suggested that glycotransferases in the goblet cell secretory apparatus or mucin glycan degrading enzymes in the colon may be responsible for the different glycan profiles.

### 3.5. Membrane-Associated Mucins in Colitis

**Figure 4:**
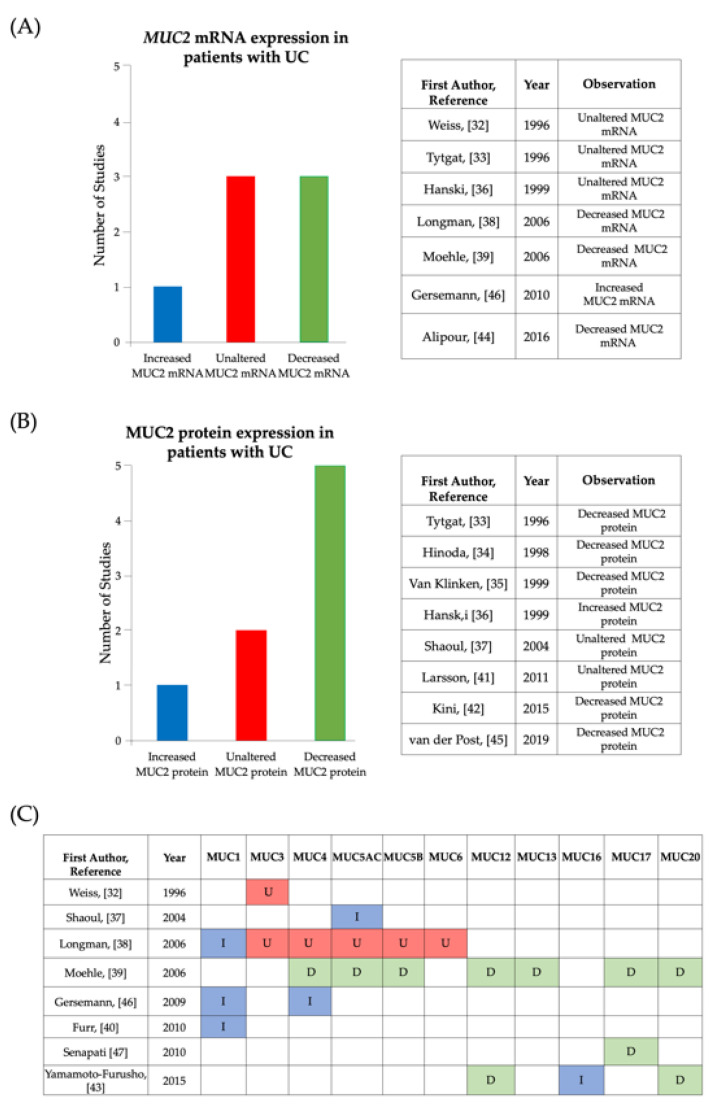
Summary of information reported in articles analyzing differences in mucin expression in active UC compared to controls. (**A**) Bar chart and table showing observations made by included studies that investigated *MUC2* mRNA expression in active UC, with controls as baseline for comparison. (**B**) Bar chart and table showing observations made by included studies that investigated MUC2 protein expression in active UC, with controls as baseline for comparison. (**C**) Table showing observations made by included studies that investigated mucin expression levels of mucins other than MUC2. Key: I-Increased expression in comparison to controls, U-Unaltered expression in comparison to controls, D-decreased expression in comparison to controls.

It has been well documented that the secretory mucin, MUC2 is the predominant mucin in the colonic mucosa of healthy individuals and individuals with UC [3,25]. A few of the included articles investigated the role of membrane-associated mucins in UC (Figure 4C). MUC1 is expressed at low levels in colonic epithelial cells [35,41]. However, Longman et al. [41] demonstrated that in samples from patients with active UC, MUC1 expression was upregulated and *MUC1* mRNA localised at crypt abscesses. Gersemann et al. [46] also reported an upregulation of *MUC1* mRNA in samples from inflamed and non-inflamed UC patient samples in comparison to controls, albeit to a lower extent than in CD patient samples. Additionally, Furr et al. [40] determined that this altered expression of MUC1 in the colon is exclusive to IBD, as aberrant MUC1 was not detected when investigations were carried out with non-IBD abnormal colonic biopsy specimens (from patients with celiac disease). As observed for *MUC2* mRNA expression, Weiss et al. [32] reported that *MUC3* mRNA expression levels were unaltered during active UC. Both Weiss et al. [32] and Longman et al. [38] showed that *MUC3* mRNA is expressed at the colonic surface epithelium and colonic crypts, despite inflammation. Longman et al. [41] also showed that *MUC4* mRNA was strongly expressed in the cytoplasm of colonic epithelia in UC and control samples. Further to this Gersemann et al. [46] recorded an upregulation of MUC4 in UC patient samples. Yamamoto-Furusho et al. [43] conducted a study aiming to understand the potential role of MUC12, MUC16 and MUC20 in UC, since very few studies have investigated these mucins in UC. The study found that, *MUC12* mRNA expression was significantly reduced in active UC in comparison to UC in remission and controls and this reduction was associated with the perturbation of the colonic mucosa. When Moehle et al. [39] investigated *MUC12* mRNA expression in UC, they recorded concordant results. Yamamoto-Furusho et al. [43] also reported an increase in *MUC16* mRNA in active UC and UC in remission compared to controls. However, this study did not find an association between MUC16 expression and the clinical presentation of UC. On the contrary, Yamamoto-Furusho et al. [43] found a link between the expression of MUC20 in UC and remission, the expression of MUC20 was increased in samples from patients with UC in remission in comparison with patients with active UC and controls. The study suggested that the increase in MUC20 mRNA expression is part of a protective mechanism. The expression of MUC17 in UC was investigated by both Moehle et al. [39] and Senapati et al. [47]. Moehle et al. [39] observed a downregulation of MUC17 mRNA in patient UC samples in comparison to controls. Through immunohistochemical analysis, Senapati et al. [47] observed a significant reduction in the expression of MUC17 protein in UC patient samples in comparison to controls, this study also examined differences in the localisation of MUC17. Senapati et al. [47] recorded loss of expression of MUC17 within the colonic crypts of UC samples and sparse expression on the surface colon columnar cells, which was markedly different to the controls which had strong expression of MUC17 within the crypt epithelial cells. The study [47] also determined that this difference in MUC17 expression was associated with inflammation but was not restricted to patients with UC, as they recorded a similar reduction and presentation of MUC17 expression within colonic tissue resected from patients with Ischaemic Colitis.

### 3.6. MUC5AC in Colitis

MUC5AC is a secretory mucin normally expressed in the gastric mucosa [37]. Since it has been documented that MUC5AC is expressed in goblet cells of the small intestine when inflammation is present, Shaoul et al. [37] aimed to determine whether MUC5AC is expressed in the colonic mucosa of individuals with UC and reported that *MUC5AC* mRNA co-expressed with *MUC2* mRNA in goblet cells in UC samples. On the other hand, Longman et al. [41] could not detect *MUC5AC* mRNA in UC samples.

## 4. Discussion

**Figure 5:**
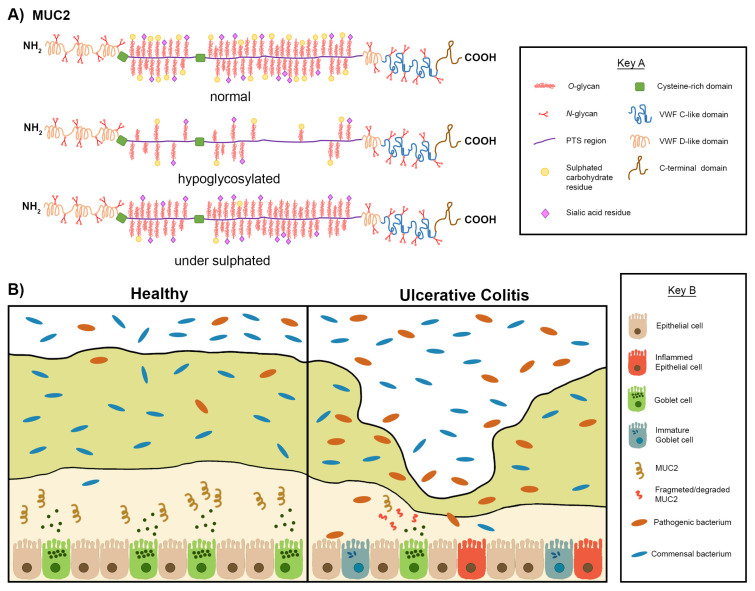
Graphical summary describing the various and dynamic changes in mucin structure and the mucus layer in relation to Ulcerative Colitis (UC). (**A**) Schematic representations of the mucin MUC2 with domain features explained in Key A. In UC aberrant post translational modifications can lead to hypoglycosylation of MUC2. Hypoglycosylation results in the PTS region being exposed, therefore an unstable form of MUC2 is produced. Mucin glycans can also be under sulphated as a result of aberrant post translation modifications in patients with UC. These aberrations leading to conformational pressure of the MUC2 structure make MUC2 more susceptible to mucin degradation by pathogenic bacteria which are present in the colonic milieu during UC. (**B**) Schematic representation of the mucosal layer in healthy individuals and patients suffering from UC with features with explained in Key B. There are two sublayers of mucus, the outer layer and the inner layer, mucins are produced by goblet cells in the epithelia and most are expressed within the inner layer of colonic mucus as presented on the left-hand side. Mucins in the adherent layer of healthy individuals form interactions which aid to limit the contact between luminal contents and the epithelia. Mucin degradation and mucin synthesis are in equilibrium in healthy individuals. In UC studies have reported a decrease in the number of goblet cells and subsequently a decrease in mucin secretion as shown on the right-hand side of 5B. Studies have also reported an increase in the number of immature goblet cells (poorly differentiated) which leads to the production of unstable mucins (hypoglycosylated and/or under sulphated). The mucosal membrane also becomes perturbed resulting in the infiltration of the luminal microbiota into the inner mucus layer. Increased contact between epithelial cells and bacteria results in inflammation as shown.

This systematic review was conducted to investigate the relationship between mucins and UC. This review provides detailed analysis of the current published information pertaining to the expression of mucins in the colonic mucosa of individuals with UC. The majority of studies reviewed presented evidence of alterations in the expression of colonic mucins in patients with UC at either the mRNA or protein level and this is summarized in Figure 5. One of the aims of this review was to gain a clearer understanding of the role of MUC2 in the development of UC and to explore the role of MUC2 in the regulation of the protective colonic mucosal barrier. Conflicting results were presented by studies with regards to *MUC2* mRNA expression (Figure 4A). Out of the seven studies that investigated *MUC2* mRNA expression, 14% (*n* = 7) recorded an increase in *MUC2* mRNA in UC whilst 43% (*n* = 7) of studies observed a decrease and 43% (*n* = 7) observed no significant difference in *MUC2* mRNA. Likewise, data extracted from studies assessing MUC2 protein expression presented mixed findings (Figure 4B). Whilst 63% (*n* = 8) reported a reduction, 25% (*n* = 8) of the studies did not notice a change in expression between UC samples and control samples and 13% (*n* = 8) recorded an increase in MUC2 protein expression. It is important to note that the methods used for detection of *MUC2* mRNA and MUC2 protein expression varied amongst the studies (Table 2) and this may be indicative of the recorded differences in findings. The differences in findings may also be reflective of the differences in cohort demographics (age, gender, ethnicity) or geographical region (studies from 9 different countries were included) since different genetic variants associated with UC have been found in different geographical regions [23].

It is also important to note that UC is a heterogenous disease therefore, dysregulation of mucins may differ from one subset of patients to another [20]. UC is heterogenous in terms of the type and level of inflammation observed, with subsequent consequences on disease severity [9,49]. Despite this, perturbation of the mucosal membrane and penetration of the luminal microbiota into the inner mucus layer is a common hallmark of UC, highlighting the importance of mucosal integrity in intestinal health [3,11,49]. van der Post et al. [45] showed that the increase in microbes at the colonic mucosa during active UC reduced the secretory activity of goblet cells responsible for the production of MUC2. Further to this, it has been shown that several different spatially distinct goblet cell phenotypes are altered in UC [50]. How changes in goblet cell number and phenotype, and subsequent alternations to mucin expression, may impact UC disease severity has yet to be fully elucidated. Differences in study methodology may have also had an impact, for example the nature of controls included in studies varied (Table 1). This may have influenced the observations that were recorded for control samples and consequently the differences in mucin expression and activity reported between controls and patient samples. The histological scoring indices used to categorise patients as having active UC or UC in remission also differed. Some of the scoring indices used included Matts inflammation score, TrueLove and Richards Index, Mayo Score and Sandborn histological score (Table 1).

Additionally, Hanski et al. [36] were the only study to use the Remmele and Stegner immunoreactive score to assess MUC2 protein levels and were the only study to detect an increase in MUC2 protein levels in UC. The lack of a standardised method for the interpretation of immuno-histochemical data for the identification of MUC proteins is highlighted here. The finding from Hanski et al. [36] may indicate that the immunoreactive score is either a more precise method for interpretation or is more susceptible to producing false positives.

Nevertheless, making conclusions solely based on the direction of expression (increases or decreases), is insufficient. The mechanisms behind these findings must be broken down to understand how MUC2 expression is regulated in UC. For example, Tytgat et al. [33] suggested that MUC2 is regulated at the translational level and during active UC *MUC2* mRNA is inefficiently translated whereas, Hanski et al. [36] suggested that changes in MUC2 protein expression resulted from inefficient post-transcriptional modification of MUC2. Both of these findings may be related and should be investigated further since, post-transcriptional modifications have an impact on the subsequent translation of mRNAs to proteins. Although Tytgat et al. [33] reported a reduction in MUC2 protein and Hanski et al. [36] reported an increase, the suggestions made by both studies highlight the importance of understanding how the process of protein production influences mucin structure and in turn the impact of mucin structure on mucin function. For example, aberration of post-translational modifications of mucins in UC such as glycosylation and sulphation have an impact on mucin degradation [35,37,41]. Two of the analysed studies, Shaoul et al. [37] and Larsson et al. [41] described altered glycan profiles of mucins in UC. Further to this, it has been shown that hypoglycosylation and abrupt termination of glycosylation of MUC2 in UC exposes the PTS domain [2,25]. The absence of glycans in the PTS domain makes MUC2 more susceptible to unregulated degradation by proteolytic enzymes. These alterations in mucin structure and subsequent degradation have an impact on the level of mucin expression and could contribute to the thinning of the colonic mucosa in UC [2,25].Moreover, the presence of a thinner colonic mucosa may lead to increased infiltration and contact of luminal microbial components with the colonic epithelia, which then drives an inflammatory response.

Although several mucins other than MUC2 have been identified in the healthy colon, this review highlights that the information about the role of these mucins in UC is still very limited. An understanding of the differences in expression and structure of these mucins in the colon of healthy individuals and individuals with UC may enhance our understanding of the role of mucins in UC. Research has shown that a large proportion of the genes encoding secreted mucins, such as MUC2 are clustered on the same chromosomal locus, 11p15 [10]. Therefore, studies directed at assessing the ancestral relationship between mucins, the influence of genetics on mucin transcription and how this directs their function and interaction with each other during UC may be important. One of the included articles, Shaoul et al. [37], described the co-expression of MUC2 and MUC5AC in goblet cells of samples from UC patients. Interestingly, Forgue-Lafitte et al. [51], one of the studies excluded due to the lack of controls, conducted a comparative study of patients with UC and patients with colon cancer. In this study they found that high levels of MUC5AC in UC were associated with inflammation and the presence of pre-cancerous lesions. Since chronic UC can develop into colon cancer, they propose MUC5AC as a surveillance biomarker for the progression of UC. This study highlights the importance of understanding the role of mucins in UC.

Goblet cell depletion has been observed in the colonic mucosa of UC patients and alterations in MUC2 expression have been associated with this depletion of goblet cells and the thinning of the colonic mucosa [25]. The influence of goblet cells on the colonic mucosa and the expression of mucins was assessed in a number of the included articles [33,34,37,41,42,45,46]. It was noted that microbial challenge of goblet cells in active UC led to attenuation of their secretory action and played a role in the structural weakening of the colonic mucosa [45]. The study carried out by van der Post et al. [45] emphasises the importance of enhancing our understanding of the fate of mucins during UC. The microbial challenge is a model of the microbial penetration of the mucus layer that leads to the perturbation of the colonic mucosal epithelia in UC. It has been shown that some pathogenic bacteria are able to infiltrate the host through attachment to mucins, therefore the impact of bacterial colonisation must be evaluated when considering these changes in the mucosal response and goblet cell function [2,3,18,52,53]. The increased microbial contact with colonic epithelia in UC is thought to lead to overstimulation of goblet cells resulting in exhaustion of goblet cells and subsequently reduced secretion of mucus [24,45]. This reduction of mucus coupled with an increasing penetration of bacteria has been associated with an increase in inflammation in UC [24]. Consequently, this can lead to more aberrant differentiation of goblet cells, and as stated by Hinoda et al. [34] this results in reduced production of mucins. The findings from these articles collectively highlight the complexity of the interactions and mechanisms that drive the perturbation of the colonic epithelia, leading to aberrant function and differentiation of goblet cells. However, it is evident that the impact of bacterial colonisation and inflammation on goblet cell function and in turn mucin production and expression needs to be studied further. Kini et al. [42] and Gersemann et al. [46], focus on the importance of understanding the pathways and factors involved in the initiation of goblet cell differentiation and the impact of this on mucin synthesis and consequent expression.

Unfortunately, meta-analysis could not be included in this review because during the data extraction process, it was established that articles used varying methods and units to measure mucin expression. Furthermore, some of the included studies used histological analysis rather than quantitative data to make conclusions about mucin expression. It was concluded that because of the large differences in reporting style used across the studies, conducting a meta-analysis by trying to convert data presented to the same format may introduce procedural bias. However, the data presented in this review still provides evidence that mucin expression is altered during UC and highlights the importance of further studies for clarifying the role of mucins in the pathogenesis of UC. Although the role of mucins in the pathogenesis of UC cannot be ignored, it must be noted that the aetiology of IBD is multifactorial and this review provides just a snapshot of the full picture. It is therefore important to design investigations that explore the interactions between the mucins and other factors within the colonic milieu, for example models can be designed to investigate these interactions at the molecular level. Collaborative multinational studies investigating the role of mucins in UC patients would also enable a larger number of samples from a wider population of patients (varied geographical distributions) with varying presentations of UC to be analysed. Furthermore, larger studies will provide more precise results as differences in demographics can be accounted for and findings will be easier to generalise. Future studies should ideally be longitudinal, so changes can be monitored throughout the course of the disease although it is recognised that there are significant logistical challenges to such an approach. It is also important to identify how changes in mucin expression and structure may be functionally implicated in disease progression. Therefore, further investigations applying a combination of Single Cell RNA Sequencing (scRNAseq) and pathway enrichment analysis can be geared towards determining upstream factors responsible for the observed changes in mucin expression, activity and structure and the downstream consequences. Chromatin immunoprecipitation (ChIP) assays have also been shown to be a good method for investigating how mucin gene expression is regulated [54]. To complement this, structural changes in the mucus layer should be observed by comparing samples from patients with different severities of disease with samples from healthy controls. Microscopy imaging techniques such as spatial light interference microscopy (SLIM) can be used to determine changes in mucus thickness and mass spectrometry coupled with proteomics can be used to deduce differences that may exist in mucin structure [54]. Employing these strategies to study the changes in mucin expression, activity and structure in patients with UC, may inform the development of new therapeutic targets for the colonic mucosal barrier. Undeniably, this review highlights the necessity of more studies exploring the relationship between UC and mucin expression, activity and structure, since only 44% of the included studies were published in the last 10 years (Table 1). Looking forward, these studies are required to determine whether the changes in mucin expression in patients with UC are significant and to clarify which changes in mucin expression, activity and structure occur.

## 5. Conclusions

In conclusion, the studies assessed in this systematic review identified alterations in mucin activity, expression, synthesis and structure in colonic biopsy samples from patients with UC. Some of the reviewed publications provided evidence of the reduction in mucin expression levels but this was not reproduced in all investigations. However, this may be explained by differences in the assessment of mucin expression in the included studies. Using the findings of the included articles, this review also drew a connection between the changes in mucin structure that occur as a result of UC and mucin degradation. Further studies are required to explore whether the observed alterations of mucins in UC precede pathogenesis or occur during pathogenesis. Nevertheless, the present systematic review, provides a basis to inform further investigations which may enhance our understanding of how mucin regulation is involved in the pathogenesis of UC.

## Figures and Tables

**Table 1:** Summary of the study design and sample characteristics for each of the included articles.

Identification			Population			Methods Used	
First Author, Reference	Year	Country	Control Samples	Nature of Control Samples	Patient Samples	Diagnosis-Disease Severity/Scoring Indices	Study Design
Weiss, [32]	1996	USA	1	Resected tissue from patient with colon diverticular disease	8	Method of Diagnosis/ Scoring Indices used are not stated	Case–control study
Tytgat, [33]	1996	Netherlands	7	Negative history of IBD (colon appeared normal endoscopically and histologically)	Active UC: 6, Inactive UC (remission): 6	Truelove and Richards Index	Case–control study
Hinoda, [34]	1998	Japan	14	Normal colonic tissue obtained post-mortem (from autopsies)	31	Matts Score	Case–control study
Van Klinken, [35]	1999	Netherlands	12	Tissue resected from patients diagnosed with Irritable Bowel Syndrome, hyperplastic polyps or diverticulosis but with negative history of IBD	Active UC: 13, Inactive UC (remission): 12	Truelove and Richards	Case–control study
Hanski, [36]	1999	Germany	13	Histologically normal mucosae samples	70	Matts Score (degree of inflammation), Remmele and Stegner (immunoreactive score for immunohistochemistry)	Case–control study
Shaoul, [37]	2004	Japan	5	Samples taken from study groups histology library (original source of controls is not stated)	5	Method of Diagnosis/Scoring Indices used are not stated	Case–control study
Longman, [38]	2006	UK	17	Tissue resected from patients undergoing elective colorectal resection surgery but with negative history of IBD	40	Truelove and Witts Criteria	Case–control study
Moehle, [39]	2006	Germany	14	Tissue resected from patients following colonoscopy (8 with no tissue abnormalities; remaining samples from patients with diverticulitis, polyposis coli, lymphoid tissue-lymphoma, carcinoma, diverticulosis, eosinophilic colitis, infectious colitis	Active UC: 14, Inactive UC (remission): 5	Diagnosis based on clinical features and radiologic/endoscopic findings	Case–control study
Gersemann, [46]	2009	Germany	21	Tissue resected from patients undergoing routine colonoscopy (specific reasons for colonoscopy are not outlined)	Active UC: 25Inactive UC: 15	Method of Diagnosis/Scoring Indices used are not stated	Case–control study
Furr, [40]	2010	USA	21	Tissues randomly resected from patients undergoing colonic biopsy but with negative history of IBD	14	Diagnosis based on medical records	Case–control study
Senapati, [47]	2010	USA	12	Patients undergoing colonoscopy whose colonoscopic exams/histology were normal	25	Diagnosis based on medical records	Case–control study
Larsson, [41]	2011	Sweden	25	Tissue resected from patients during colonoscopy for polyp surveillance, investigation of anaemia or rectal bleeding (normal colonic mucosa, no signs of inflammation)	Active UC: 15, Inactive UC (remission): 13	Sandborn’s histological activity score	Case–control study
Kini, [42]	2015	India	12	Tissue resected from patients with IBS undergoing routine colonoscopy or during polyp surveillance colonoscopy	22	Truelove and Witts (clinical disease severity), Ulcerative Colitis Disease Activity Index (endoscopic severity)	Case–control study
Yamamoto-Furusho, [43]	2015	México	30	Tissue resected from patients during colonoscopy for polyp surveillance/screening and evaluation for anaemia (normal colonic mucosa, no signs of inflammation)	Active UC: 20, Inactive UC (remission): 20	Mayo score (clinical and endoscopic activity evaluation), Riley score (histological activity evaluation)	Case–control study
Alipour, [44]	2016	Canada	12	Tissue resected from patients with Irritable bowel syndrome, benign polyps, chronic diarrhoea but negative history of IBD and no signs of inflammation	10	Paris Classification and Paediatric Ulcerative Colitis Activity Index (PUCAI)	Case–control study
van der Post, [45]	2019	Sweden	47	Tissues resected from patients during colonoscopy; patients with polyps, diverticulosis but with negative history of IBD	Active UC: 36, Inactive UC (remission): 28	Mayo score (clinical and endoscopic activity evaluation) and/or Sandborn’s histological activity score	Case–control study

**Table 2:** Summary of the purpose for articles included for analysis and key findings.

First Author, Reference	Purpose of Study	MUC Gene Investigated	Measuring MUC Expression	Key Findings
Weiss, [32]	To investigate effect of inflammation on expression of mucin genes at cellular level	MUC2, MUC3	In-situ hybridisation with RNA probes	MUC2 and MUC3 expression in colonic mucosa is independent of inflammation
Tytgat, [33]	To study regulation of MUC2 expression in patients with UC compared with controls	MUC2	MUC2 precursor quantified by SDS-PAGE, total MUC2 by dot blot, in-situ hybridisation with RNA probes to quantify MUC2 mRNA	Inefficient translation of MUC2 mRNA may lead to the reduction in MUC2 synthesis observed in active UC
Hinoda, [34]	To determine if MUC2 protein expression is altered in UC	MUC2	MUC2 protein detected by Immunohistochemistry	Decreased MUC2 protein production and expression in active UC is associated with undifferentiated goblet cells
Van Klinken, [35]	To determine whether there are alterations in MU2 sulphation and secretion in active UC	MUC2	Analysis and quantification of total MUC2 using SDS-PAGE and dot blotting	Absolute amount of MUC2 secreted is decreased and mucins are under-sulphated in active UC
Hanski, [36]	To study alterations in MUC2 expression in UC patient colonic tissue	MUC2	MUC2 protein detected by immunohistochemistry and MUC2 mRNA detected using in-situ hybridisation	Increase in MUC2 protein detection in UC samples may be related to reduction in post transcriptional modification
Shaoul, [37]	To investigate alterations in expression and distribution of MUC2, MUC5AC, trefoil factor 1 (TFF1) in UC	MUC2, MUC5AC	PAS/Alcian blue immunohistochemistry	Immature (poorly glycos-ylated) MUC2 is expressed in UC colonic mucosa depleted of goblet cells
Longman, [38]	To investigate alterations in the expression of mucin genes and trefoil peptide genes in UC	MUC1-6	Immunohistochemistry and in-situ hybridisation	MUC1 expression upregulation is associated with severe UC and there is a reduction of MUC2 expression in UC
Moehle, [39]	To characterize changes in mucin expression and identify allelic variants of MUC genes in UC	MUC1-20	Affymetrix DNA-microarray analysis and RT-PCR	MUC12 mRNA expression is downregulated in UC and is independent of inflammation
Gersemann, [46]	To understand the mechanisms involved with goblet cell differentiation and mucin production in IBD	MUC1, MUC2, MUC4	RT-PCR	Impairments in goblet cell differentiation factor induction in UC correlates with a reduction in mucin synthesis.
Furr, [40]	To determine whether MUC1 expression is altered in IBD	MUC1	Immunochemistry using anti-MUC1 anti-bodies	Overexpression and hypoglycosylation of MUC1 observed in a subset of UC patients
Senapati, [47]	To determine the subcellular localization of MUC17 in colonic mucosa and to determine whether MUC17 expression is altered in IBD and neoplastic diseases.	MUC17	Immunohistochemistry using anti-MUC17 polyclonal antibody	MUC17 expression is reduced in colonic mucosa of UC patients
Larsson, [41]	To determine whether MUC2 glycosylation is related to degree of mucosal inflammation in UC	MUC2	SDS-PAGE used to identify and quantify MUC2	Alterations in MUC2 glycosylation are associated with inflammation
Kini, [42]	To determine whether alterations occur in colonic stem cells during the pathogenesis of UC and to determine the impact of such changes on goblet cell development and proteins synthesized by goblet cells	MUC2	H&E, Alcian blue and PAS immunohistochemistry staining to detect MUC2 protein	A reduction in MUC2 protein within the lower colonic crypt precedes inflammation.
Yamamoto-Furusho, [43]	To determine if MUC12, MUC16, MUC20 expression changes in UC	MUC12, MUC16, MUC20	MUC gene expression measured using RT-PCR and MUC protein expression measured using immunohistochemistry	MUC16, MUC20 expression increase in UC and increase in MUC20 was associated with remission of UC
Alipour, [44]	To assess whether mucosal barrier defects are prerequisites to UC	MUC2	Fluorescence in-situ hybridisation (FISH) and immunofluorescence	Reduction in mucin-containing goblet cells and mucin production in UC patients compared to controls
van der Post, [45]	To investigate compositional alterations that occur at the adherent mucus layer in UC	MUC2	Absolute quantification of MUC2 using Skyline (V.3.6.0.1) following mass spectrometry	Reduction of MUC2 in active UC associated with exhaustion of secretory response of goblet cells to microbes

Periodic acid–Schiff/Alcian blue (PAS/Alcian blue), Haematoxylin and eosin (H&E), Sodium Dodecyl Sulphate-Polyacrylamide Gel Electrophoresis (SDS-PAGE), Real Time Polymerase Chain Reaction (RT-PCR).
